# OCT proves that vitreomacular adhesion is significantly more likely to develop vision-threatening retinal complications than vitreomacular separation

**DOI:** 10.1186/s12886-020-01416-x

**Published:** 2020-04-22

**Authors:** Ding-Ying Liao, Jorn-Hon Liu, Yu-Ping Zheng, Huei-Wen Shiu, Jian-Ming Wang, Hsiao-Ming Chao

**Affiliations:** 1 Department of Ophthalmology, Second Affiliated Hospital, Xi’an Jiaotong University, Xi’an, Shaanxi China; 2 Department of Ophthalmology, Cheng Hsin General Hospital, Taipei, Taiwan; 3 Institute of Pharmacology, School of Medicine, National Yang-Ming University, Taipei, Taiwan; 4 Department of Chinese Medicine, School of Chinese Medicine, China Medical University, Taichung, Taiwan; 5 Department of Ophthalmology, Taipei Medical University-Shuang Ho Hospital, Ministry of Health and Welfare, Taipei, Taiwan; 6 Department of Ophthalmology, Shin Kong Wu Ho-Su Memorial Hospital, Taipei, Taiwan

**Keywords:** Vitreomacular adhesion, Vitreomacular separation, Incidence, Macular hole, Vitreomacular angle

## Abstract

**Background:**

SD-OCT is becoming commonplace in everyday practice. Vitreomacular adhesions (VMAs) are being more routinely diagnosed. Predictive studies to the natural course of VMA are thus clinically significant. Spectral domain-optical coherence tomography (SD-OCT) was presently utilized to analyze the incidence of floaters, the complete vitreomacular separation or VMA, the VMA complication, the vitreomacular angle (VMAng), and the complication mechanism.

**Methods:**

Monthly SD-OCT was performed on patients with/without symptomatic floaters. OCT allowed VMA and vitreomacular separation to be compared. The incidence was assessed applying one-tailed Fisher’s exact tests. The VMAngs between the inner retina and posterior hyaloid were measured, and the complication mechanism was studied using OCT image. For macular hole (MH), pre- and/or post-operative best corrected visual acuities (BCVAs; LogMAR), refractions and photoreceptor conditions were also evaluated.

**Results:**

Totally, 124 eyes were included; there were 116 eyes with VMA and 8 eyes with vitreomacular separation. Considering the percentages over 124 eyes, floaters were present in 14.5% of enrolled eyes (=18/124), consisting of 12.9% of eyes with VMA (16/124) and 1.6% of eyes with vitreomacular separation (2/124). Moreover, there were twelve eyes (9.7%) with VMA-associated vision-threatening complications, including MH (*n* = 8; 6.5%), retinal detachment (RD; *n* = 2; 1.6%), vitreomacular traction (VMT; *n* = 1; 0.8%) and macular pucker (MP; *n* = 1; 0.8%). Eyes with initial VMA had a significantly greater possibility of complications than eyes with initial vitreomacular separation (*p* = 0.03). Among these eyes with MH (*n* = 8), the pre-operative BCVA (LogMAR) was 1.1 ± 0.5, which was insignificantly (*p* = 0.35) improved to 0.8 ± 0.7 post-operatively. The VMAng of VMA eyes with MHs was 24.2 ± 24.9° (*n* = 8). The critical VMAng was 13.3°.

**Conclusions:**

A minority of eyes with VMA or vitreomacular separation had floaters. Moreover, the use of SD-OCT could identify vision-threatening sequelae, namely MH, RD, MP and VMT, and this was significantly more frequent in eyes with VMA than in eyes with complete vitreomacular separation. Therefore, SD-OCT might be a useful way of identifying either identity, and evaluating VMA-associated complications. Whether VMA eyes with MH (*n* = 8) that have a VMAng greater than critical VMAng have a greater likelihood of tangential traction and subsequent MH needs further investigation.

## Introduction

The recent development of spectral domain-optical coherence tomography (SD-OCT) has allowed practitioners to obtain more detailed information on vitreomacular interface diseases [1]. As indicated by previous reports [2, 3], vitreomacular adhesion (VMA) can result in a variety of vision-threatening complications; these include macular hole, retinal detachment, vitreomacular traction and macular pucker. According to the SD-OCT-based investigation of Duker et al. [3], VMA is defined as a perifoveal vitreous detachment that still has remaining vitreomacular attachment within a 3-mm radius of the fovea (defined arbitrarily) without detectable distortion of the foveal contour. In contrast, vitreomacular separation is presently given a definition of complete vitreous detachment from the defined fovea without residual VMA. With the aid of SD-OCT, vitreomacular interface conditions such as VMA are able to be more precisely identified despite the transparency of the vitreous [1]. However, conventional ocular/vitreoretinal examinations still need to be routinely used at present in order to compensate for the scan length limitation of OCT, which is centered on the fovea [4].

Normally, OCT demonstrates that vitreomacular separation starts as an initial perifoveal vitreous detachment with remaining VMA. In the following years, as it progresses, there is contemporary liquefaction of the vitreous gel, which eventually results in complete vitreomacular separation [5]. VMA is compared with complete vitreomacular separation as part of the present study.

As indicated by Kakehashi et al. [6], floaters only seem to be present in a minority of eyes with posterior vitreous detachment; thus, misdiagnosis is possible when patients are included in this diagnosis only if there is an acute sign (a Weiss ring) and/or symptoms such as floaters [6–9]. Therefore, the regular use of SD-OCT might be a useful approach to identify VMAs, when there is a lack of signs and/or symptoms [6]. What is more, a long-term follow-up would seem to be necessary in order to examine patients specifically for the presence of various defined vision-threatening complications that are known to be associated with VMA.

The current study compares the frequency of visual dysfunctional complications present in SD-OCT proved VMA eyes with those eyes that had complete vitreomacular separation eyes, with or without signs/symptoms, and also with the results obtained in previous studies of acute symptomatic posterior vitreous detachment eyes [2, 3, 7, 8, 10]. The hypothetical mechanism in which a macular hole is related to the occurrence of VMA is also investigated by linking the results obtained to the eye’s vitreomacular angle. Pre- and post-operative best corrected visual acuities as well as preoperative ocular refraction values, and postoperative macular photoreceptor conditions are evaluated, too.

## Methods

### Inclusion and exclusion criteria of this study

The Institutional Review Board at Cheng Hsin General Hospital, Taipei, Taiwan [approval number: (607)106–15; Supplementary file 1] gave permission for this chart review study. During the baseline examination by SD-OCT (Spectralis, Heidelberg Engineering, Heidelberg, Germany) between January 1st 2016 and July 31st 2017, one-hundred and twenty-four eyes (one or two eyes per individual) with either vitreomacular separation (*n* = 8) or VMA with perifoveal vitreous detachment (*n* = 116) were selected and followed up by SD-OCT monthly. The scan type was a volume (cube) scan. Additionally, scan length [6 × 6 mm (20°× 20°)], scan density (25 line scan/20°× 20°) and resolution [3.9 μm (digital)] were set as indicated. The incidence of floaters was presently assessed. Patients were excluded if their medical records indicated that they were not asked “are you suffering from floaters in your vision?”. The incidence rates for various complications related to either of the defined conditions were also evaluated. To evaluate the complication incidence, the cases with defined complications were also pinpointed after an initial SD-OCT was able to prove diagnosis. Observation of the disease course was carried out in order to establish how the two defined entities are related to the various defined complications. Furthermore, patients were excluded if they had any of the following previous ocular diseases, there were glaucoma, proliferative diabetic retinopathy, uveitis, ocular trauma, vitreous hemorrhage as well as *aforementioned* complications. In addition, patients that did not undergo regular follow-up were excluded.

### SD-OCT examination to identify VMA or vitreomacular separation

The medical records included whether or not patients had an onset of VMA or vitreomacular separation related symptoms, such as floaters, a distorted image and blurred vision. In addition to SD-OCT, every enrolled eye received conventional ocular/vitreoretinal examinations by indirect ophthalmoscope [6], and slit-lamp biomicroscopy with the help of a contact lens to detect a range of signs such as the presence of a retinal break, a retinal atrophic hole and lattice degeneration.

When VMA was present, in contrast to unilateral posterior hyaloid detachment (J-shaped pattern), the partial posterior vitreous might be detached both nasally and temporally, but persistent attachment could be observed to be maintained at the fovea, which resulted in a V-shaped pattern [11].

### Measurement of the vitreomacular angle

For clinical practicability, the vitreomacular angle of the present cases were measured as follows. Two lines were drawn from the foveal center: one horizontal line along the inner retina and the other oblique line along the separated posterior hyaloid. The angle between the inner retinal surface and posterior hyaloid was then directly measured using the On-Screen Protractor Program (Minimum Java Runtime Environment version 1.7, Gnu’s Not Unix *General Public License version 3*); the largest angle for VMA, with either V-shaped or J-shaped posterior hyaloids, of an eye that received both vertical and horizontal OCT scans was defined as the vitreomacular angle of the examined eye. The directly measured critical vitreomacular angle was 13.3°, which is considerably lower than that 45.0° of the study of Tsai et al. [12]. The above vitreomacular angle was retrieved by utilizing the point on the receiver operating characteristics curve with a minimum value of [(1 − sensitivity)^2^ + (1 − specificity)^2^] for macular hole in all of the selected eyes.

### Statistics

Under the present hypothesis that there are more complications with VMA [8], the one-tailed Fisher’s exact test was used to analyze the enrolled eye results obtained during the present study. This allowed an analysis of whether VMA shows a higher incidence of defined vision-threatening retinal complications than vitreomacular separation. All data collected during this study were analyzed using *Statistical Package for the Social Sciences 20.0* (Statistical Package for the Social Sciences, Incorporation, Chicago, *Illinois*, United States of America). The data are expressed as mean ± standard deviation. The unpaired Student’s *t*-test was used when comparing two independent groups. A probability value of < 0.05 was considered significant.

## Results

**Fig. 1:**
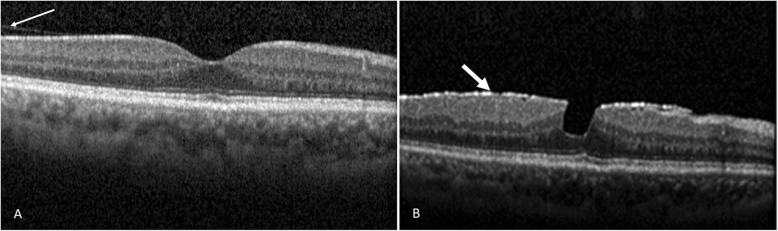
*The initial and subsequent SD-OCT findings.* There was vitreous detachment temporal to the fovea (**a**; as indicated by a thin arrow; initial OCT image) with nasal vitreomacular adhesion in Case 2; the subsequent OCT image (**b**) demonstrates the development of macular pucker (indicated by a white arrow) with a lamellar hole 3 months later. Abbreviation: SD-OCT, spectrum domain-optical coherence tomography

**Fig. 2:**
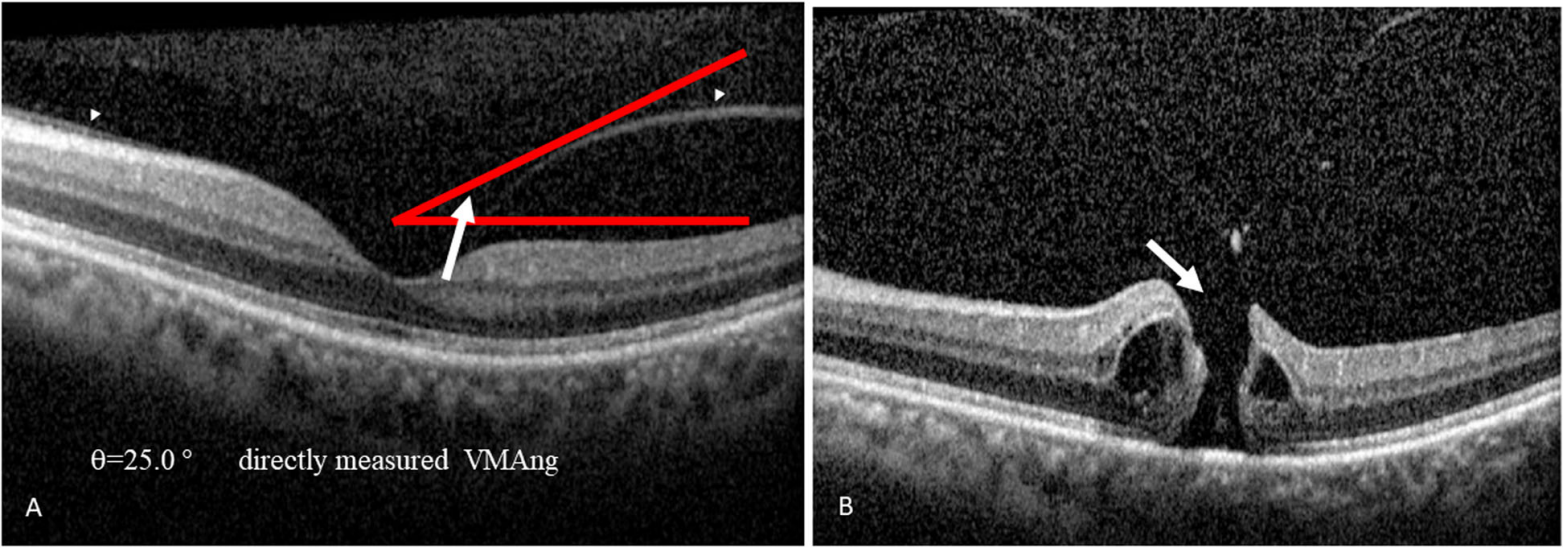
*The initial and subsequent SD-OCT findings.* The Heidelberg Spectralis SD-OCT retinal finding of Case 4 (**a**) demonstrated an initial V-shaped vitreomacular adhesion (VMA; indicated by two separate arrow heads). The angle was formed by drawing two lines, namely one horizontal line along the inner retina and the other oblique line along the posterior hyaloid. The largest angle out of “the temporal and nasal” angles (V-shaped VMA) was defined as the VMAng of the examined eye that received both vertical and horizontal OCT scans. The VMAng directly measured was calculated using the On-Screen Protractor Program (A). The VMAng (A: 25°) is indicated by a white arrow. The retinal image of Case 4 (**b**) upon a subsequent visit revealed the formation of macular hole (indicated by a white arrow) 11 months later. Abbreviations: SD-OCT, spectrum domain-optical coherence tomography; VMAng, vitreomacular angle

**Fig. 3:**
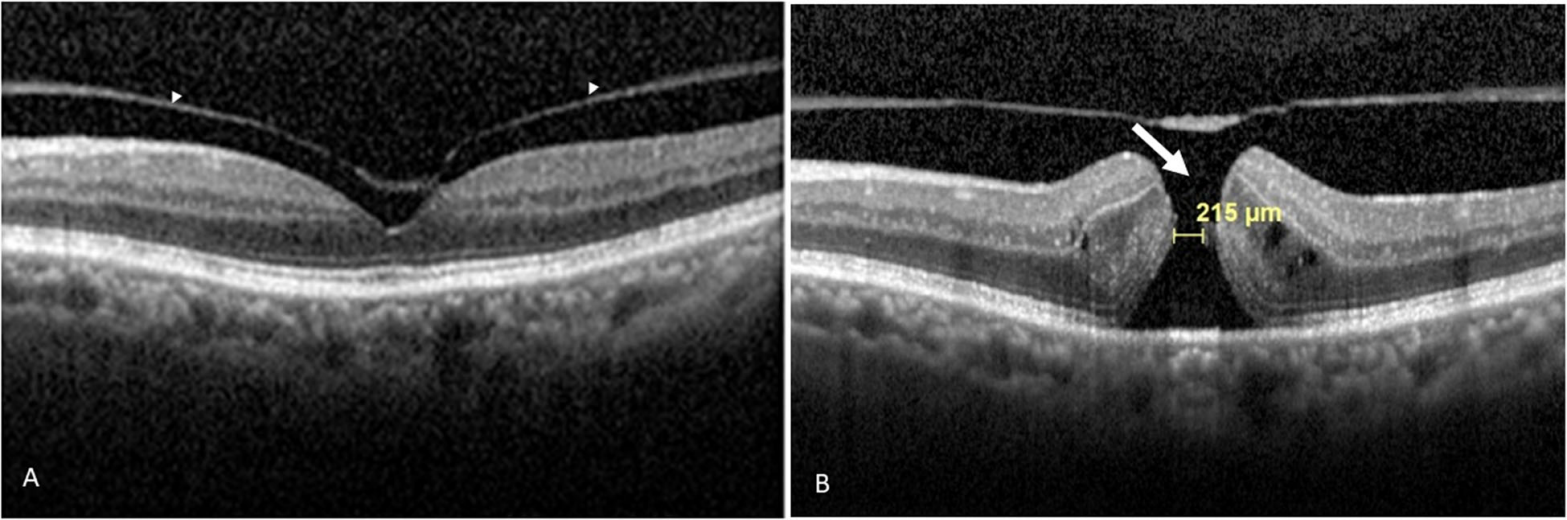
*The initial and subsequent SD-OCT findings.* The Heidelberg Spectralis SD-OCT retinal finding of Case 5 (**a**) showed an initial V-shaped vitreomacular adhesion (indicated by two separate arrow heads). The retinal image of Case 5 (**b**; a hole with a diameter of 215 μm) upon a subsequent visit revealed the formation of a macular hole (indicated by a white arrow) 3 months later. Abbreviations: SD-OCT, spectrum domain-optical coherence tomography

**Fig. 4:**
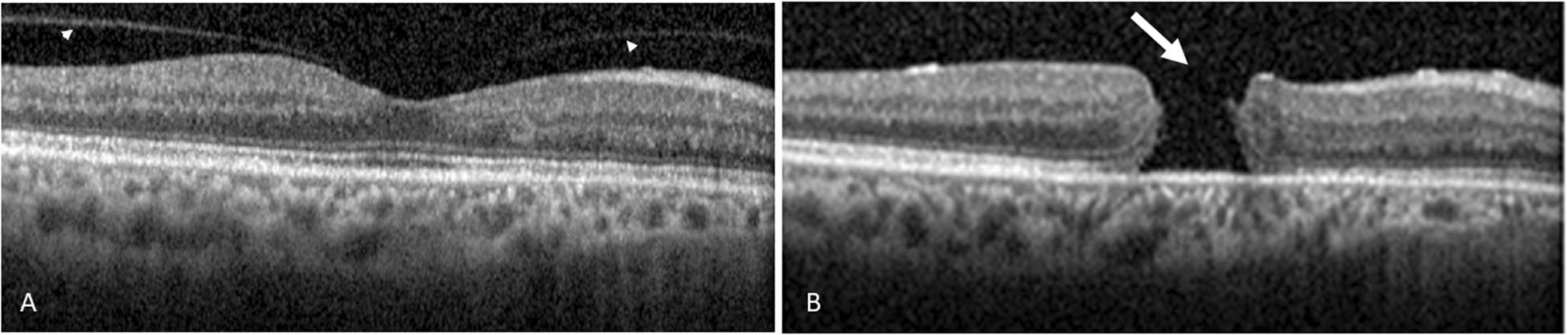
*The initial and subsequent SD-OCT findings.* The Heidelberg Spectralis SD-OCT retinal finding of Case 12 with a V-shaped vitreomacular adhesion (**a**; indicated by two separate arrow heads; initial OCT image) was revealed; the subsequent image (**b**) showed the formation of full-thickness macular hole (indicated by a white arrow) 3 months later. Abbreviations: SD-OCT, spectrum domain-optical coherence tomography

The relevant SD-OCT findings and baseline data were as follows. A total of 116 out of 124 eyes (93.5%) were found to show VMA; whereas, 8 eyes (6.5%) were found to show complete vitreomacular separation. The follow-up duration was 11.07 ± 5.24 months (range, 0.8 to 19 months). Symptomatic floaters were present in 14.5% of the studied eyes (=18/124), specifically 12.9% of eyes with VMA (16/124) and 1.6% of eyes with vitreomacular separation (2/124). Among the 124 eyes studied, the numbers of male and female subjects were 70 eyes (mean age, 59.9 ± 10.0 years) and 54 eyes (mean age, 54.8 ± 13.2 years), respectively. Among eyes with vitreomacular separation, the numbers of males and females were 6 eyes and 2 eyes, respectively. Among the VMA eyes, the numbers of male and female were 64 eyes and 52 eyes, respectively. The mean age of all patients of the enrolled eyes was 57.6 ± 11.6 years; while that of those patients with VMA eyes or vitreomacular separation eyes were 57.4 ± 11.8 and 60.5 ± 8.0 years, respectively. During the follow-up period, vision-threatening retinal complications (Tables 1 and 2) occurred in twelve eyes (9.7%), these being macular hole in eight eyes (6.5%; Figs. 2b, 3b and 4b); retinal detachment in two eyes (1.6%; Table 2); vitreomacular traction in one eye (0.8%; Supplementary file 2B) and macular pucker in one eye (0.8%; Fig. 1b). These sequelae occurred in eyes with an initial VMA and were not found in eyes with an initial vitreomacular separation. Among the initial VMA eyes with subsequent complications of macular hole (*n* = 8; Tables 1 and 2), there were six eyes that displayed a V-shaped VMA (for example: Figs. 2a, 3a and 4a); for comparison, there were two eyes that displayed nasal or temporal VMA with respective temporal or nasal perifoveal vitreous detachment. Moreover, among the two eyes with sequelae of retinal detachment (Tables 1 and 2), one showed a V-shaped VMA, while the other one demonstrated nasal VMA with temporal perifoveal vitreous detachment. Only one eye with the complication of vitreomacular traction (Tables 1 and 2) revealed a V-shaped VMA (Supplementary file 2A). Additionally, one nasal VMA eye with a sequela of macular pucker (Tables 1 and 2) showed temporal perifoveal vitreous detachment (Fig. 1v). The clinical features, SD-OCT findings, the onsets of signs/symptoms across the twelve eyes with an initial VMA and the presence of subsequent vision-threatening retinal sequelae were recorded (Table 2). The vitreomacular angles (see **Measurement of the vitreomacular angle** in the **Methods** section) of the eight patients with VMA and consequent sequelae involving macular holes are significantly (*p* = 0.04) higher at 24.2 ± 24.9° (Table 2) when compared to those of patients without adverse complications (*n* = 104; 13.6 ± 13.1°). For example, in Fig. 2a, the vitreomacular angle was 25°.

**Table 1:** Initial SD-OCT findings and the incidence of visual dysfunctional sequelae for VMA and VMS during the follow-up

Initial OCT Findings	No.	VMT	MP	MH	RD	Adverse Sequelae
VFA eyes (%)	116 (93.5)	1(0.8)	1(0.8)	8.(6.5)	2(1.6)	12(9.7)
V-shaped(%)	76	1	0	6	1	8
N/T adhesion (%)	40	0	1	2	1	4
VMS eyes (%)	8 (6.5)	0(0)	0(0)	0(0)	0(0)	0(0)
Total enrolled eyes (%)	124(100)	1.(08)	1(0.8)	8(6.5)	2(1.6)	12(9.7)

Abbreviations: *SD-OCT* spectrum domain optical coherence tomography; *VMA* vitreomacular adhesion; *VMS* vitreomacular separation; *VMT* vitreomacular traction; *MP* macular pucker; *MH* macular hole; *RD* retinal detachment; *N/T* nasal or temporal

**Table 2:** Clinical features, SD-OCT findings and new onsets of signs/symptoms among patients with initial vitreomacular adhesion that progressed into vision-threatening sequelae.

Case number	age range (yrs)	Sex	Eye	SD-OCT Findings of VMA^a^	Cx (**VMAng)**^b^	Management	VA (Pre~post-op)	New Onset Signs/Symptoms
01	70~79	1	OD	partial detachment temporal to fovea	FTMH^c^ (**82.9**°)	VT+Peel	2.0~1.3^d^	N. fundus/VA↓
02	50~59	2	OD	partial detachment temporal to fovea	MP (1.2°)	VT+Peel	0.3~0.2	N. fundus/floaters
03	40~49	2	OD	partial detachment temporal to fovea	RD (8.0°)	VT+SB+SO	1.3~0.7	LD & RB/flashes
04	60~69	2	OS	V-shaped	FTMH^c^ (**25.0**°)	VT+Peel+SB	0.7~0.0^d^	Retina hole/distortion
05	60~69	2	OS	V-shaped	FTMH^c^ (**14.9**°)	VT+Peel+SB+SO	0.8~1.3^d^	RB/VA↓
06	60~69	2	OS	V-shaped	FTMH^c^ (**25.0**°)	VT+Peel	0.8~0.4^d^	N. fundus/VA↓
07	40~49	1	OD	V-shaped	FTMH^c^ (**19.3**°)	VT+Peel	1.0~2.0^d^	foveoschisis/VA↓
08	40~49	1	OS	V-shaped	FTMH^c^ (**2.8**°)	VT+Peel	0.7~0.2^d^	N. fundus/Distortion
09	20~29	1	OD	V-shaped	RD (6.2°)	VT+SB+SO	1.3~2.0	LD & RB/VA↓
10	30~39	1	OS	partial detachment nasal to fovea	FTMH^c^ (**13.2°**)	VT+Peel	1.3~0.7^d^	foveoschisis/VA↓
11	70~79	2	OS	V-shaped	VTM (9.2°)	VT+Peel	0.3~0.2	Macula cyst/VA↓
12	70~79	1	OD	V-shaped	FTMH^c^ (**10.5°**)	VT+Peel	1.3~0.5^d^	N. fundus/flashes

^a^SD-OCT findings of vitreomacular adhesion (VMA) which was divided into 2 types, namely V-shaped VMA (see Case 4 in Fig. 2a) as well as nasal or temporal perifoveal vitreous detachment with respective temporal or nasal VMA (see Case 2 in Fig. 1vs).

^b^The VMAng was directly measured (underlined and bolded for FTMH; e.g. Case 4: 25.0°; Please also refer to the section **Measurement of the vitreomacular angle** of the **Methods**) by the On-Screen Protractor Program (Minimum JRE version 1.7, GNU GPL v3).

^c^After above vitreoretinal surgeries that were respectively given to 8 cases of FTMH, there were 5 cases (62.5%: Case 1, 4, 6, 8 and 12) of closed hole, 1 case (Case 10) of sealed hole and 2 cases (Case 5 and 7) of unclosed hole. Postoperatively, there were 5 cases (62.5%: Case 1, 5, 7, 8 and 10) with macular photoreceptor inner segment-outer segment (IS-OS) disruption and 3 cases (Case 4, 6 and 12) without IS-OS disruption.

^d^Furthermore, the pre-operative best corrected visual acuities (BCVAs; LogMAR) were, though not significantly, improved from 1.1±0.4 to 0.8±0.7. Distortion is defined as distorted image.

Abbreviations: *SD-OCT* spectrum domain optical coherence tomography; *Cx* complication; *yrs* years; *VMAng* vitreomacular angle; *VA* (Pre~post-op); *BCVA* (Pre~post-op: pre-~post-operative visit); *LogMAR* logarithm of the minimum angle of resolution; *FTMH* full-thickness macular hole; *MP* macular pucker; *RD* retinal detachment; *VMT* vitreomacular traction; *LD* lattice degeneration; *RB* retinal break; *VT* pars plana vitrectomy; peel, internal limiting membrane peeling; *SB* scleral buckle; *SO* silicon oil tamponade.

The present hypothesis that there would be significantly more complications with VMA, was confirmed using a one-tailed Fisher’s exact test (*p* = 0.03). Specifically, eyes with VMA have a significantly greater possibility of developing defined visual dysfunctional retinal complications compared to eyes with vitreomacular separation (Table 3). After vitreoretinal surgery (Table 2) to treat eight cases of full thickness macular hole, there were five cases (62.5%: Case 1, 4, 6, 8 and 12) of closed hole, one case (Case 10) of sealed hole and two cases (Case 5 and 7) of unclosed hole. Postoperatively, there were five cases (62.5%: Case 1, 5, 7, 8 and 10) with macular photoreceptor inner segment-outer segment disruption and three cases (Case 4, 6 and 12) without inner segment-outer segment disruption. Furthermore, the pre-operative best corrected visions (logarithm of the minimum angle of resolution) were 1.1 ± 0.5, and these were improved to 0.8 ± 0.7, although this improvement was not significantly (*p* = 0.35; *n* = 8).

**Table 3:** The incidence of vision-threatening retinal complications after VMA and VMS evaluated by one-tailed Fisher’s exact test

Subtype	No. of adverse Cx	No. of none adverse Cx	*p value*
VMA	12	104	0.03
VMS	0	8	0

95% confidence interval is 0.69–0.85

Abbreviations: Cx Complication; *p* Probability; *VMA* vitreomacular adhesion; *VMS* vitreomacular separation

The mean refractions (diopter) of all of the enrolled eyes (− 1.7 ± 5.0; *n* = 124) were listed as follows: vitreomacular separation (1.0 ± 2.5; n = 8), VMA (− 1.9 ± 5.0; *n* = 116), VMA without complication (− 1.5 ± 4.1; *n* = 104), VMA with complications (− 5.4 ± 9.7; *n* = 12) and VMA with macular holes (− 7.0 ± 11.7; *n* = 8). There was a significant (*p* = 0.01) refractive difference between VMA without complications (*n* = 104) and with complications (*n* = 12). Furthermore, a significant (*p* = 0.003) difference also existed between VMA without complications (*n* = 104) and VMA with complications of macular hole (*n* = 8). Moreover, there was an almost significant difference (*p* = 0.05) between VMA (− 1.9 ± 5.0; *n* = 116) and vitreomacular separation (1.0 ± 2.5; *n* = 8).

## Discussion

It has been reported that the development of perifoveal vitreous detachment with remaining VMA, or with complete vitreomacular separation, rarely occurs in patients younger than 50-year-old [3, 5]. This is also the case in the present study with the mean age of the subjects being 57.6 years old. In a prospective study, Uchino, Uemura and Ohba [5], found that the percentage of incomplete to complete posterior vitreous detachment was 85.1% vs. 14.9%. This contrasts to a degree with the present study, where the percentage of VMA to that of vitreomacular separation is 93.5% vs. 6.5%. Another prospective study of Kakehashi et al. [6], using slit-lamp biomicroscopy, found the frequencies of the various types of posterior vitreous detachment (*n* = 200 eyes) were 51.0% (=102/200) of complete type, 36.0% (=72/200) of incomplete type, and 13.0% (=26/200) with no Weiss ring eyes. Moreover, in both cases, the majority of eyes with a complete glial ring (59.0% = 60/102) and the majority of eyes with an incomplete glial ring (68.0% = 49/72) had no floaters. What is more, 93.0% (24/26) of the eyes without a Weiss ring had no symptomatic floaters, either [6]. This agrees with the present results, which indicated that floaters are only present in a minority (14.5%) of selected eyes. Therefore, a diagnosis of VMA or vitreomacular separation might easily be missed when studying outpatients who are recruited only because of the presence of an acute sign (Weiss ring) and/or symptomatic floaters, which has been the situation in previous studies [6, 7]. Therefore, monthly SD-OCT is very likely presently be useful and assist with assessing the development of the dynamic VMA and helping to determine the incidence of VMA associated retinal complications such as macular hole.

### The development of VMA or vitreomacular separation analyzed using OCT

Compared to the results of Abdolrahimzadeh et al. (a frequency of 85% for incomplete posterior vitreous detachment) [10], 93.5% of the eyes in the present study presented with VMA (116 out of 124). Ito et al. [13] stated that posterior vitreous detachment starts from the perifoveal region, next it continues to the fovea and finally, it develops into complete posterior vitreous detachment. Nevertheless, how the exact dynamic evolution of VMA takes place needs to be further investigated.

### VMA related vision-threatening retinal sequelae proved by SD-OCT

As implied by previous reports [3, 12, 14–21], VMA can be complicated by a variety of defined visual dysfunction retinal diseases. The present results support the hypothesis that the incidence of defined retinal complications among VMA eyes seems to be significantly greater than that among vitreomacular separation eyes (Tables 1, 2 and 3; Figs. 1, 2, 3 and 4; Supplementary file 2). This agrees with the widely accepted concept that the former have persistently stronger adhesions that pull at the fovea [3, 12, 14–21], and induce detrimental complications. By way of contrast, the latter group seems to have lost this strong traction [3, 12, 17, 22]. Thus, accurate diagnosis, long term observation and appropriate management needs to be emphasized when treating patients with VMA associated adverse sequelae. As mentioned earlier, SD-OCT seems to be a clinically useful and an easily accessible approach that is able to detect various specific vitreomacular interface abnormalities that might not be observed by ultrasonography and indirect ophthalmoscopy [3, 12, 17, 22].

### The observations on refraction values in relation to VMA-induced complications

As mentioned at the final paragraph of the **Results**, in contrast to the defined low hyperopia (1.0 ± 2.5) of vitreomacular separation, low myopia (− 1.5 ± 4.1) or high myopia (− 5.4 ± 9.7/− 7.0 ± 11.7) seems to be related, respectively, to VMA without complications and VMA with complications/macular holes. A further larger scale study is needed in order to investigate the role of the high myopia in the development of VMA-induced complications/macular holes.

### Vitreomacular traction

Vitreomacular traction seems to be able to produce tangential (horizontal) [12, 22] and anteroposterior (vertical) traction [12, 22] that causes foveal conformational changes and subsequent visual dysfunctional symptoms such as blurred vision, floaters/flashes and/or a distorted image. In a twenty-year cross-sectional study, namely the Beaver Dam Eye Study [21], it was found that the incidence of vitreomacular traction was 1.6% among all eyes with maculae scanned by SD-OCT. In contrast, the incidence of vitreomacular traction was presently estimated to be 0.8% (1 out of 124 eyes; Table 1). Classic vitreomacular traction could be divided into two types: V-shape and J-shape [11]; while, another method subclassified vitreomacular traction into focal (vitreomacular attachment diameter ≤ 1500 μm) and broad subtypes (> 1500 μm) [23]. Kishi et al. demonstrated that the margin of the 1500 μm foveal region had a firm vitreoretinal adhesion [24]. As recommended by Bottos et al. [25], the classification of vitreomacular traction, based on the diameter of the adhesion, appears to be more precisely than that based on its morphology.

### Macular holes

Over the past two decades, the literatures [3, 12, 22, 26–32] available on the incidence of adverse retinal disorders such as macular hole that are associated with VMA has been limited. Furthermore, the findings are quite variable due to differences in a range of factors; these include the study design (cross sectional or longitudinal; prospective or retrospective), the diagnostic tools used (ultrasound, OCT, etc) and the different criteria utilized to define the classification and diagnosis of VMA or vitreomacular separation (with or without acute symptoms) [2–4, 8, 12, 22, 27]. Additionally, a recent retrospective multicenter study [27] has reported that full thickness macular holes occurred in 7 out of 168 eyes (4.2%) with vitreomacular traction. This differs to some extent to the present retrospective study in which the incidence of full thickness macular holes was 6.5% (8 out of 124 eyes). Compared to the present results, it has been suggested that VMA and vitreomacular traction appear to be two different entities in view of the difference in the incidence (6.5% vs. 4.2%) of full thickness macular hole [27]. There are also differences in SD-OCT-based findings, namely without or with interruption of all foveal retinal layers and distortion of the foveal contour (normal depression vs. dome-shaped elevation; see also Supplementary file 2) [2, 3, 11, 12, 27]. In contrast to the vitreomacular traction associated idiopathic full thickness macular hole that occurs sometimes in the presence of foveal detachment due to vertical traction [12], none of the present eight VMA eyes with subsequent full thickness macular hole complications showed foveal detachment. Gass [28] has postulated that premacular vitreous tangential contraction might lead to a macular hole. This hypothesis regarding tangential vitreoretinal traction has been replaced by a newer theory, which suggests that macular holes are the result of anteroposterior vitreofoveal traction in the perifoveal area [12, 22, 29, 30]. However, the postulation of Gass seems to explain better the present finding, which is that six out of eight eyes (Tables 1 and 2) displayed V-shaped VMAs (Figs. 2a, 3a and 4a) and that this state might have induced tangential traction resulting in the complication, specifically, full thickness macular hole. Compared to VMA with unilateral hyaloid membrane separation, V-shaped VMAs perhaps indicate a bilateral pull with greater traction force. This might then be more likely to induce macular hole formation. However, this hypothesis needs further investigation. In a prospective report of Johnson, Van Mewkirk and Meyer [32], it was shown by the SD-OCT that perifoveal vitreous detachment was the initial pathogenesis associated with grade 1–2 macular hole (*n* = 26). Moreover, persistent vitreofoveolar adherence, namely VMA [3], was evident in 18 eyes of those eyes with defined macular hole. This evidence [32] also strongly supports why eyes with VMA in the present study have a significant greater possibility of ocular complications, such as macular hole (*n* = 8), than eyes with vitreomacular separation. In contrast to the above, the study of Carrero [8] was prospective and the recruited eyes had acute symptoms; this might have contributed to his finding that macular holes were not found.

Tsai et al. [12] and Theodossiadis et al. [22] have reported that the vitreomacular angles between the posterior vitreous membrane and the horizontal lines of the retina (inner retinal surface [12] or retinal pigment epithelium [22]) are proportionally associated with the severity of vitreomacular traction [22, 31, 33]. Their findings [12. 22] seem to support the present results whereby the mean vitreomacular angle (Fig. 2; Table 2) of the eight eyes with VMA and consequent sequelae, i.e. macular holes, was significantly (*p* = 0.04) higher at 24.2°, with possible stronger traction force, compared to those of patients without adverse complications (*n* = 104; at 13.6°). This is not inconsistent with a recent prospective study [31] where out of fifty one cases of VMA (2%), one case (Case 2) with an initial vitreomacular angle 27° nasally or 20° temporally [31] [vs. 31.2° nasally or 24.6° temporally (measured by the present measurement method)] showed an evolved vitreofoveal separation associated with a highly suspected complicated macular hole. This was in spite of an intact external limiting membrane (presumably due to healing process), 10 days after the last examination in the VMA stage. To determine whether VMA eyes with a vitreomacular angle more than the present critical vitreomacular angle of 13.3° (mean) have a greater possibility of associated macular hole complication requires a larger scale investigation. On the other hand, Tsai et al. [12] and Spaide et al. [26] have suggested that the wider the diameter of the VMA, the stronger the traction exerted on the fovea. However, the adhesion diameters (mean: 239.0 ± 52.5 μm) in the present study are somewhat difficult to be definitively measured, for example the broad typed VMA [3] nasal to the fovea presented in Case 2 (Fig. 1a). V-shaped vitreomacular traction has been reported to lead to tractional macular hole [25]. This seems also to be the case in our study and the above study when there are initial V-shaped VMAs, specifically for six out of 8 eyes with complicated macular holes in the present study (Tables 1 and 2) and for the above mentioned Case 2 with a suspected complicated macular hole in Figure 6 (middle) of a recent publication [31]. Classification of VMA induced macular hole, based on the morphology, seems to be at present the most practical approach, rather than one based on the diameter of the adhesion.

### Rhegmatogenous retinal detachment

Most incomplete posterior vitreous detachments with retinal detachment are likely to be diagnosed as being the rhegmatogenous type [14]. Abdolrahimzadeh et al. [10] suggested that some peripheral vitreoretinal adhesions were not easily visible clinically and that retinal breaks could occur by sudden traction. It is reasonable to postulate that the posterior vitreous membrane might be pulled from its residual peripheral attachment sites, which will then cause retinal tears and this could then extend into the rhegmatogenous retinal detachment. As demonstrated by the present study, because the incidence of retinal detachment is 1.6% (2 out of 124 eyes), retinal detachments are likely to be due to a new onset of peripheral retinal lattice degeneration, which is then followed by a vitreous traction-induced retinal break. This seems to be the situation with Case 3 and 9. However, the ability to detect the peripheral lesions, such as a break, is beyond the scan length limitation of SD-OCT.

There are other limitations to the present retrospective study. First, the number of enrolled eyes is not that large (*n* = 124). Moreover, if the patient is not followed up for long enough, is lost to follow-up, switches to another consultant, or moves to another hospital for further treatment, this individual will be lost to the investigator and this lack of contact means that vision-threatening sequelae affecting the patients will remain unidentified. As mentioned, given that the study excluded patients who were not asked about floaters or who were unable to receive SD-OCT examinations due to urgent operations for diseases such as macular hole, there might also be the risk of selection bias.

Apart from those limitations, the present study does indicate that there is a greater proportion (93.5%) of VMA than vitreomacular separation among these enrolled eyes. Moreover, as compared to vitreomacular separation, VMA does have a significantly greater possibility of progression into various defined vision-threatening retinal complications. Whether eyes with VMA might undergo horizontal (tangential) traction, with this playing a role in the pathogenesis of a consequent macular hole, needs further evaluation. What is more, whenever general ophthalmologists identify a patient with VMA, it might be useful that regular follow-ups are carried out, especially by means of tests such as monthly SD-OCT, if this is available, that targets the vitreomacular interface. Moreover, other conventional fundus examinations that can detect peripheral retinal lesions, including breaks or lattice degeneration, need to be carried out, if possible with the pupils being fully dilated. Prompt referral to a vitreoretinal specialist is warranted when any defined visual dysfunction retinal complication occurs.

## Conclusions

Nearly 90% of the enrolled eyes in this study had VMA. Defined vision-threatening sequelae (9.7% = 12/124), including macular hole, occur significantly more frequently in eyes with VMA than when eyes have vitreomacular separation. Monthly SD-OCT for each eye of VMA or vitreomacular separation with/without symptoms/signs from the initial visit onwards might be considered useful in order to clarify vitreomacular interface conditions. VMA eyes that have a greater mean vitreomacular angle (24.2°) than the presently defined present critical angle of 13.3° seems to have a higher probability of developing a subsequent full thickness macular hole. Moreover, the findings of V-shaped VMAs being present in majority of eyes (6/8 eyes) with macular holes might be regarding an indication of bilateral horizontal (tangential) traction. This might play a role in the pathogenesis of the consequent macular hole. In the long-term, it might be useful to closely monitor VMA using SD-OCT in order to allow the timely discovery and management of any subsequent visual dysfunctional complications despite the length limitation associated with SD-OCT detection.

## Supplementary information

**Additional file 1. ***An official agreement for waiving the requirements to obtain Informed Consent.* The Institutional Review Board at Cheng Hsin General Hospital, Taipei, Taiwan [Approval No: CHGH-IRB (607)106–15] agreed this retrospective chart review study.

**Additional file 2. ***The initial and subsequent spectrum domain optical coherence tomography findings.* In Case 11, an initial V-shaped vitreomacular adhesion (A) evolved into vitreomacular traction (B) with a macular cyst 6 months later.

**Additional file 3. ***Cover letter.* Inside were included responses to the Editor, Professor Haoyu Chen as well as the Reviewers, Professor Peiquan Zhao and Professor Stanley Chang.

**Additional file 4. ***Measurement of the vitreomacular angle.* In Case 4 with vitreomacular adhesion and V-shaped hyaloid membranes, while measuring the VMangles, the temporal angle differed from the VMangle on the nasal side of the foveal attachment. There were temporal and nasal angles respectively calculated from horizontal (25° and 5°; A) and vertical OCT scans (24° and 4°; B). The largest angle, i.e. 25°, was selected as the VMangle as shown in the Fig. 2a. The measurement of the VMangle mentioned in the 3rd section **Measurement of the vitreomacular angle** of the **Methods** was thus the same as that shown in the Fig. 2a.

## Data Availability

The data shown and/or analyzed during the present investigation are available from the corresponding author upon reasonable request.

## Abbreviations

VMAs: Vitreomacular adhesions
VMS: Vitreomacular separation
SD-OCT: Spectral domain-optical coherence tomography
VMAng: Vitreomacular angle
MH: Macular hole
BCVAs: Best corrected visual acuities
LogMAR: Logarithm of the minimum angle of resolution
RD: Retinal detachment
VMT: Vitreomacular traction
MP: Macular pucker
