# Supplementary material for: OCT proves that vitreomacular adhesion is significantly more likely to develop vision-threatening retinal complications than vitreomacular separation
Source: BMC Ophthalmol. 2020 Apr 22;20:163. doi: 10.1186/s12886-020-01416-x (PMC7178608; doi:10.1186/s12886-020-01416-x)
Supplement: Supplementary file 1 — Additional file 1. An official agreement for waiving the requirements to obtain Informed Consent. The Institutional Review Board at Cheng Hsin General Hospital, Taipei, Taiwan [Approval No: CHGH-IRB (607)106–15] agreed this retrospective chart review study. [file 12886_2020_1416_MOESM1_ESM.pdf]

振興醫療財團法人振興醫院人體試驗委員會

臨床試驗計畫同意函

CHGH-IRB 編號：(607)106-15 新案
計劃編號：
計畫名稱：不完全後玻璃體剝離：盛行率與黃斑裂孔臨床相關性
計畫主持人：趙效明醫師、劉榮宏醫師
執行機構：振興醫院
審查通過版本：
計畫書：Ver 2.0, 2017/10/13
中文計畫摘要：2017.10.16
本案同意免除知情同意過程

同意函有效期：自 106 年 11 月 1 日至 107 年 11 月 1 日止

上述計劃符合本院人體試驗委員會作業基準之簡易審查案件，已於 106 年 11 月 1 日經初審委員簡易審核通過同意執行，並將提近期委員會議追認。若在委員會議未獲追認通過，本委員會將另行通知，主持人需依照人體試驗委員會決議，配合辦理。有關計劃主持人的職責、義務、及注意事項均詳列於背面，請參閱並遵守。

主任委員

中華民國 106 年 10 月 28 日

Cheng Hsin General Hospital Permission of Clinical Trial

Date:28 Oct. 2017

CHGH-IRB No: (607)106-15
Protocol No:
Protocol Title: Incomplete Posterior Vitreous Detachment: Prevalence and Clinical Relevance of Macular Holes
Principle Investigator: Hsiao-Ming Chao, Jorn-Hon Liu
Institute: Cheng Hsin General Hospital
Version:
Protocol: Ver 2.0, 2017/10/13
Chinese Synopsis : 2017.10.16
We agree to waiver of the requirements to obtain Informed Consent

The above research protocol fit in with the expedited review cases in CHGH IRB SOP010. Although it has already been initially approved on 1 Nov. 2017 and valid through 1 Nov. 2018, we still ought to verify the decision at our next board meeting. If it fails, we will inform the principle investigator immediately and the principle investigator should follow our final decision. About the essential duties, obligations and responsibilities of the principal investigator (PI), please refer to the back page.

人體試驗委員會
E-mail:irb@chgh.org.tw
TEL:(02)28264400
Ext:7707

Chairman
Institutional Review Board

本委員會組織與執行皆符合 ICH-GCP 規範及赫爾辛基宣言之精神
This Committee has been organized and operated in conformance with ICH-GCP requirements and the essence of Declaration of Helsinki.
