# Supplementary figures and images for: OCT proves that vitreomacular adhesion is significantly more likely to develop vision-threatening retinal complications than vitreomacular separation
Source: BMC Ophthalmol. 2020 Apr 22;20:163. doi: 10.1186/s12886-020-01416-x (PMC7178608; doi:10.1186/s12886-020-01416-x)

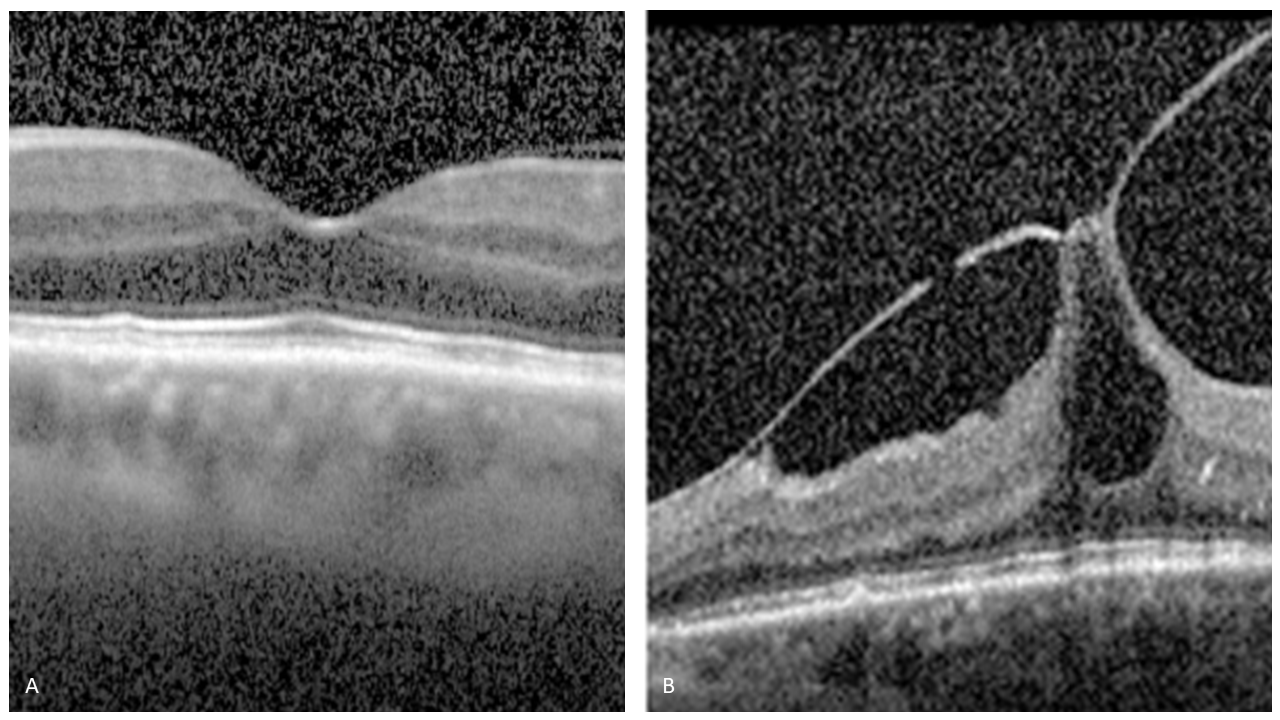

Supplement: Supplementary file 2 — Additional file 2. The initial and subsequent spectrum domain optical coherence tomography findings. In Case 11, an initial V-shaped vitreomacular adhesion (A) evolved into vitreomacular traction (B) with a macular cyst 6 months later. [file 12886_2020_1416_MOESM2_ESM.tif]

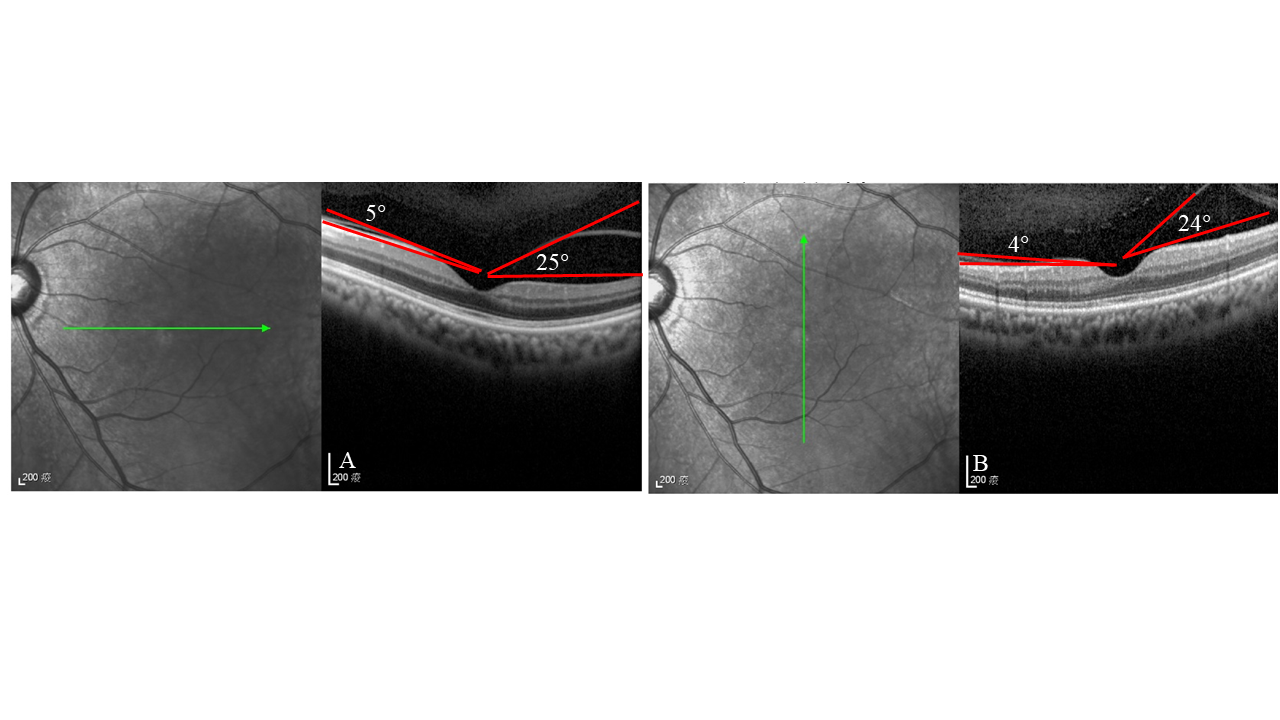

Supplement: Supplementary file 4 — Additional file 4. Measurement of the vitreomacular angle. In Case 4 with vitreomacular adhesion and V-shaped hyaloid membranes, while measuring the VMangles, the temporal angle differed from the VMangle on the nasal side of the foveal attachment. There were temporal and nasal angles respectively calculated from horizontal (25° and 5°; A) and vertical OCT scans (24° and 4°; B). The largest angle, i.e. 25°, was selected as the VMangle as shown in the Fig. 2a. The measurement of the VMangle mentioned in the 3rd section Measurement of the vitreomacular angle of the Methods was thus the same as that shown in the Fig. 2a. [file 12886_2020_1416_MOESM4_ESM.tif]
