# Supplementary material for: OCT proves that vitreomacular adhesion is significantly more likely to develop vision-threatening retinal complications than vitreomacular separation
Source: BMC Ophthalmol. 2020 Apr 22;20:163. doi: 10.1186/s12886-020-01416-x (PMC7178608; doi:10.1186/s12886-020-01416-x)
Supplement: Supplementary file 3 — Additional file 3. Cover letter. Inside were included responses to the Editor, Professor Haoyu Chen as well as the Reviewers, Professor Peiquan Zhao and Professor Stanley Chang. [file 12886_2020_1416_MOESM3_ESM.docx]

**Cover letter** (Please also refer to **Supplementary file 3**)

Dear Editor, Professor Haoyu Chen as well as Reviewers, Professor Peiquan Zhao and Professor Stanley Chang,

Please find the point-by-point responses down below.

**Reviewer reports:
Peiquan Zhao (Reviewer 1): The authors used SD-OCT proving that vitreomacular adhesion is significantly more likely to develop vision-threatening retinal complications than vitreomacular separation.
The authors enrolled 124 eyes and followed up by monthly SD-OCT. Please clarify the duration of the follow-up. Besides, are there any lost follow-up patients? This should be written in the article.**

***Response:***

*Regarding the lost follow-up patients, please refer to the final sentence of the section* ***Inclusion and exclusion criteria of this study*** *of the* ***Methods****. Regarding the follow-up duration, please refer to the 2^nd^ sentence (newly added) of the 1^st^ paragraph of the* ***Results****. The relevant answers are included with quotation marks and listed as follows.*

***Methods***

***Inclusion and exclusion criteria of this study****, final sentence*

*In addition, “patients that did not undergo regular follow-up were excluded”.*

***Results****, 1^st^ paragraph, 2^nd^ sentence*

*The “follow-up duration was 11.07±5.24 months” (range, 0.8 to 19 months).*

**Stanley Chang, MD (Reviewer 2): After reading this manuscript several times, I had difficulty understanding the materials and methods of this study. Partly this related to the description of the study since English is not the primary language of the corresponding author. It was not clear to me how the 124 eyes were selected for the study, Did the patients undergo OCT scans during the 18 month period, and only those with VMA or VMT included? Once the patient was found to have VMA or VMT, were they scanned once per month? How long was the follow-up? The measurement of the VMangle was not the same as shown on the figure. Also in some cases of measuring the VMangle, the temporal angle might differ from the VMangle on the nasal side of the foveal attachment. I found the Introduction, Methods and Results all difficult to follow in a logical fashion, and sometimes statements were made in the results that should be stated in the Discussion. Hence would recommend a significant revision of the manuscript by the authors to clarify the methods more clearly.**

***Response:***

*(i). Regarding the recruited candidates and their OCT examination interval, please refer to the 2^nd^ sentence of the section* ***Inclusion and exclusion criteria of this study*** *of the* ***Methods****. Regarding the follow-up duration, please refer to the 2^nd^ sentence (newly added) of the 1^st^ paragraph of the* ***Results****. The relevant answers and revisions are included with quotation marks and listed as follows.*

***Methods***

***Inclusion and exclusion criteria of this study****, 2^nd^ sentence*

*In the baseline examination utilizing SD-OCT (Spectralis, Heidelberg Engineering, Heidelberg, Germany) between January 1^st^ 2016 and July 31^st^ 2017, one-hundred and twenty-four eyes (one or two eyes per individual) with either “vitreomacular separation (n=8)” or “VMA with perifoveal vitreous detachment (n=116)” were selected and followed up by “monthly” SD-OCT.*

***Results****, 1^st^ paragraph, 2^nd^ sentence*

*The “follow-up duration was 11.07±5.24 months” (range, 0.8 to 19 months).*

*(ii).* *Please also refer to the* ***Supplementary file 4*** *(newly added). The relevant answers and revisions are included with quotation marks and listed as follows.*

*“****Supplementary file 4.*** *Measurement of the vitreomacular angle. In Case 4 with vitreomacular adhesion and V-shaped hyaloid membranes, while measuring the VMangles, the temporal angle differed from the VMangle on the nasal side of the foveal attachment. There were temporal and nasal angles respectively calculated from horizontal (25*° *and 5*°*;* ***A****) and vertical OCT scans (24*° *and 4*°*;* ***B****). The largest angle, i.e. 25*°*, was selected as the VMangle as shown in the* ***Figure 2A****. The measurement of the VMangle mentioned in the 3^rd^ section* ***Measurement of the vitreomacular angle*** *of the* ***Methods*** *was thus the same as that shown on the* ***Figure 2A****.”*

*Regarding the measurement of the VMangle, please refer to the following revised 3^rd^ sentence of the section* ***Measurement of the vitreomacular angle*** *of the* ***Methods*** *down below. In addition to the* ***Methods****, a significant revision of the manuscript, including* ***Introduction*** *(minor revision) and* ***Results*** *(including* ***Figure 2 legends*** *and* ***Table 2*** *‡footnote) has been made to clarify the* ***Methods*** *more clearly. Therefore, the* ***Introduction****,* ***Methods*** *and* ***Results*** *are easier to be followed in a logical fashion as required. The relevant answers and revisions are also included with quotation marks and listed as follows.*

***Methods***

***Measurement of the vitreomacular angle****, 3^rd^ sentence*

*The angle between the inner retinal surface and posterior hyaloid was then directly measured using the On-Screen Protractor Program (Minimum Java Runtime Environment version 1.7, Gnu's Not Unix* General Public License version 3*); the largest angle for “VMA, with either V-shaped or J-shaped posterior hyaloids,” of an eye that received both vertical and horizontal OCT scans was defined as the vitreomacular angle of the examined eye.*

***Figure legends***

***Figure 2,*** *3^rd^ sentence*

*The largest angle out of “the temporal and nasal” angles (V-shaped VMA) was defined as the VMAng of the examined eye that received both vertical and horizontal OCT scans.*

***Table 2****, 2^nd^ sentence, ‡footnote.*

*^‡^The VMAng was directly measured (underlined and bolded for FTMH; e.g. Case 4: 25.0°“; Please also refer to the section* ***Measurement of the vitreomacular angle*** *of the* ***Methods****”) by the On-Screen Protractor Program (Minimum JRE version 1.7, GNU GPL v3).*

*(iii). Specifically, the last two sentences at the final paragraph of the* ***Results*** *have been deleted and moved to the 3^rd^ subheading of the* ***Discussion*** *as a new paragraph, namely* ***The observations on refraction values in relation to VMA-induced complications****. Moreover, the last four sentences of the section* ***Macular holes*** *of the* ***Discussion*** *have been revised to be easily followed logically as required. The following relevant revisions are included with quotation marks, too.*

***Discussion*** *(****and Results)****, 3^rd^ subheading*

*“****The observations on refraction values in relation to VMA-induced complications*** *(a new subheading)*

*As mentioned at the final paragraph of the* ***Results****, in contrast to the defined low hyperopia (1.0±2.5) of vitreomacular separation, low myopia (-1.5±4.1) or high myopia (-5.4±9.7/-7.0±11.7) seems to be related, respectively, to VMA without complications and VMA with complications/macular holes. A further larger scale study is needed in order to investigate the role of the high myopia in the development of VMA-induced complications/macular holes.”*

***Macular holes****, 1^st^ paragraph, last four sentences.*

*In a prospective report of Johnson, Van Mewkirk and Meyer [32], it was shown by the SD-OCT that perifoveal vitreous detachment was the initial pathogenesis associated with grade 1-2 macular hole (“n=26”). “Moreover, persistent vitreofoveolar adherence, namely VMA [3], was evident in 18 eyes of those eyes with defined macular hole. This evidence [32] also strongly supports why eyes with VMA in the present study” have a significant greater possibility of ocular complications, such as macular hole (n=8), than eyes with vitreomacular separation. In contrast to the above, the study of Carrero [8] was prospective and the recruited eyes had acute symptoms; this might “have contributed” to his finding that macular holes were not found.*

***(iv).*** *Please also refer to the following* ***minor revised*** ***Abstract*** *as well as the whole* ***major revised*** ***manuscript*** *as an* ***attachment*** *in the revised submission to Editorial Manager using Word Tracking System. The following relevant revisions are included with quotation marks as well.*

***Abstract***

***Background****: SD-OCT is becoming commonplace in everyday practice. Vitreomacular adhesions (VMAs) are being more routinely diagnosed. Predictive studies to the natural course of VMA are thus clinically significant. Spectral domain-optical coherence tomography (SD-OCT) was presently utilized to analyze the incidence of floaters, “the complete vitreomacular separation or VMA, the VMA complication, the” vitreomacular angle (VMAng), and the complication mechanism.*

***Methods****: Monthly SD-OCT was performed on patients with/without symptomatic floaters. OCT allowed VMA and vitreomacular separation to be compared. The incidence “was” assessed applying one-tailed Fisher’s exact tests. The VMAngs between the inner retina and posterior hyaloid were measured“, and the complication mechanism was studied using” OCT image.* *For macular hole (MH),* *pre- and/or post-operative best corrected visual acuities (BCVAs; LogMAR), refractions and photoreceptor conditions were also evaluated.*

***Results****:* *Totally, 124 eyes were included; there were 116 eyes “with” VMA and 8 eyes “with” vitreomacular separation. Considering the percentages over 124 eyes, floaters were present in 14.5% of enrolled eyes (=18/124), consisting of 12.9% of eyes with VMA (16/124) and 1.6 % of eyes with vitreomacular separation (2/124). Moreover, there were twelve eyes (9.7%) with VMA-associated vision-threatening complications, including* *MH (n=8;* *6.5%), retinal detachment (RD; n=2; 1.6%), vitreomacular traction (VMT; n=1; 0.8%) and macular pucker (MP; n=1; 0.8%). Eyes with initial VMA had a significantly greater possibility of complications than eyes with initial vitreomacular separation (p=0.03). Among these eyes with MH (n=8), the pre-operative BCVA (LogMAR) was 1.1±0.5, which was insignificantly (p=0.35) improved to 0.8±0.7 post-operatively. The VMAng of VMA eyes with MHs was 24.2±24.9° (n=8). The critical VMAng was 13.3°.*

***Conclusions****: A minority of eyes with VMA or vitreomacular separation had floaters. Moreover, the “use” of SD-OCT “could identify” vision-threatening sequelae, namely MH, RD, MP and VMT, “and this” was significantly more frequent in eyes with VMA than in eyes with complete vitreomacular separation. Therefore, SD-OCT might be a useful way of identifying either identity, and evaluating VMA-associated complications. Whether “VMA eyes” with MH (n=8) “that have a VMAng greater than critical VMAng” have a greater likelihood of tangential traction and subsequent MH needs further investigation.*
